# Defining the Catalytic Activity of Nanoceria in the P23H-1 Rat, a Photoreceptor Degeneration Model

**DOI:** 10.1371/journal.pone.0121977

**Published:** 2015-03-30

**Authors:** Lily L. Wong, Quentin N. Pye, Lijuan Chen, Sudipta Seal, James F. McGinnis

**Affiliations:** 1 Department of Ophthalmology, University of Oklahoma Health Sciences Center, College of Medicine, and Dean McGee Eye Institute, Oklahoma City, Oklahoma, United States of America; 2 Advanced Materials Processing Analysis Center, Materials Science and Engineering, Nanoscience and Technology Center, College of Medicine, University of Central Florida, Orlando, Florida, United States of America; 3 Department of Cell Biology and Oklahoma Center for Neuroscience, University of Oklahoma Health Sciences Center, Graduate College, Oklahoma City, Oklahoma, United States of America; Justus-Liebig-University Giessen, GERMANY

## Abstract

**Purpose:**

Inorganic catalytic nanoceria or cerium oxide nanoparticles (CeNPs) are bona fide antioxidants that possess regenerative radical scavenging activities *in vitro*. Previously, we demonstrated that CeNPs had neuroprotective and anti-angiogenic properties in rodent retinal degeneration and neovascularization models. However, the cellular mechanisms and duration of the catalytic activity of CeNPs in preventing photoreceptor cell loss are still unknown. In this study, we sought to answer these questions using the P23H-1 rat, an autosomal dominant retinitis pigmentosa (adRP) model.

**Methods:**

A single dose of either saline or CeNPs was delivered intravitreally into the eyes of P23H-1 rats at 2–3 weeks of age. Retinal functions were examined at 3 to 7 weeks post injection. We quantified retinal proteins by Western blot analyses and counted the number of apoptotic (TUNEL+) profiles in the outer nuclear layer (ONL) of retinal sections. We measured free 8-isoprostanes to quantify lipid peroxidation in retinal tissues.

**Results:**

We observed increased rod and cone cell functions up to three weeks post injection. Apoptotic cells were reduced by 46%, 56%, 21%, and 24% at 3, 7, 14, 21 days, respectively, after CeNPs injection compared to saline. Additionally, reduction of lipid peroxidation in the retinas of CeNPs-treated vs saline-treated animals was detected 14 days post injection.

**Conclusions:**

We validated that CeNPs were effective in delaying loss of photoreceptor cell function in an adRP rat model. This represents the fourth rodent retinal disease model that shows delay in disease progression after a single application of CeNPs. We further demonstrated that CeNPs slowed the rate of photoreceptor cell death. We deduced that the catalytic activity of CeNPs *in vivo* in this rat model to be undiminished for at least 7 days and then declined over the next 14 days after CeNPs administration.

## Introduction

Oxidative damage due to excessive production of reactive oxygen species (ROS) is correlated with many neural degenerative diseases [1,2]. Oxidative stress, which leads to cellular dysfunction, senescence, and death, has been linked to diabetic retinopathy (DR) [3], age-related macular degeneration (AMD) [4], and glaucoma [5]. We have demonstrated that cerium oxide nanoparticles (nanoceria or CeNPs) are potent antioxidants which reduce oxidative stress, protect photoreceptor cells from degeneration and have anti-angiogenic effects in rodent models [6–11]. These bare CeNPs can cross the blood retinal barrier to protect photoreceptor neurons when introduced into the blood stream via intracardial injection in the *tubby* mouse, a photoreceptor degeneration model [7]. *In vitro*, CeNPs have catalytic activities that mimic superoxide dismutase (SOD) [12] and catalase [13], and are self-regenerating [14]. Cumulative evidence has shown that the ROS scavenging activities are due to the presence of oxygen vacancies or defects on the surfaces of these nano-sized particles and the auto-regenerative cycle between the two oxidation states, Ce3+ and Ce4+ [15]. The antioxidant and self-regenerating properties of CeNPs make them the potential therapy of choice to combat neurodegenerative diseases in the 21^st^ century because frequent dosing may be avoided. Recently, we have shown that intravitreally delivered CeNPs are rapidly taken up by retinal cells within one hour and ~50% of the injected nanoparticles are still retained in the retina after a year [16]. More significantly, our CeNPs synthesized by simple wet chemistry methods do not exhibit any toxic effect in the healthy rat retina short-, or long-term [16].

Retinitis pigmentosa (RP) is a heterogeneous group of disorders that cause rod and cone photoreceptor cell degeneration. The progressive loss of rod cells followed by cone cells ultimately leads to blindness [17]. Currently, 98 genes or loci are associated with this inherited degenerative condition. Among them 88 genes are identified, while the remaining 10 have defects in unidentified genes (RetNet Retinal Information Network). Presently, replacement of the defective gene before excessive tissue damage by gene therapy is not feasible for patients with autosomal dominant RP (adRP), although preclinical studies are underway [18,19]. We hypothesize that oxidative stress is a common node of disease progression in RP irrespective of the genetic defect and will be an ideal upstream target for therapy for this “orphan disease” class of disorders.

Mutations in the *rhodopsin* gene represent the largest percentage (30–40%) of known causes for the autosomal dominant form of RP [20]. In this condition, a single copy of the mutant gene wreaks havoc even in the presence of a normal copy of the *rhodopsin* gene. One of the point mutations discovered, P23H, (proline to histidine substitution at position 23) [21] has been recapitulated in the rat and this transgenic rat develops progressive retinal degeneration [22]. Among the three lines of P23H rats, line 1 exhibits the fastest rate of degeneration [23]. By 8 weeks of age, scotopic a-, and b-wave amplitudes were about 20%, and 60% of wildtype, respectively (Wong unpublished observations). Photopic b-wave amplitude was 56% of wildtype (Wong unpublished observations). Machida and colleagues [24] have shown that reduction in scotopic a-wave but not the b-wave correlates with the corresponding thickness reduction of the combined outer nuclear layer (ONL) and rod outer segment (ROS) as the animal ages.

Despite demonstrations of the effectiveness of CeNPs in delaying photoreceptor cell loss in two rodent models of retinal degeneration: the blindness on-demand light damage albino rat [6,25] and the recessive *tubby* photoreceptor degeneration model [7,9], little is known regarding the cellular mechanism and duration of the catalytic activity of CeNPs *in vivo*. Here, we validated the effectiveness of CeNPs in delaying the decline of rod cell function in P23H-1 rats. We showed that CeNPs prevented massive apoptosis of rod cells in a time-dependent manner after a single intravitreal injection. Because the TUNEL assay (namely, the cell death index) registers apoptotic cells at a single instant and is not accumulative for more than 6 hours, we argue that the cell death index in the retina can be used to assess the catalytic activity of CeNPs *in vivo*. Until more sensitive assays become available, the cell death index can be used as a pharmacodynamics assessment of CeNPs in the mammalian retinas. Because CeNPs effectively reduced the levels of 8-isoprostane, a reliable oxidative stress biomarker, when measured 14 days post treatment, we speculate that the reduction of rod cell apoptosis may be the consequence of improved peroxide detoxification ability of the genetically compromised rod cells after administration of a single dose of CeNPs.

## Results

### CeNPs Delayed Photoreceptor Cell Degeneration in P23H-1 Rats

**Fig 1:**
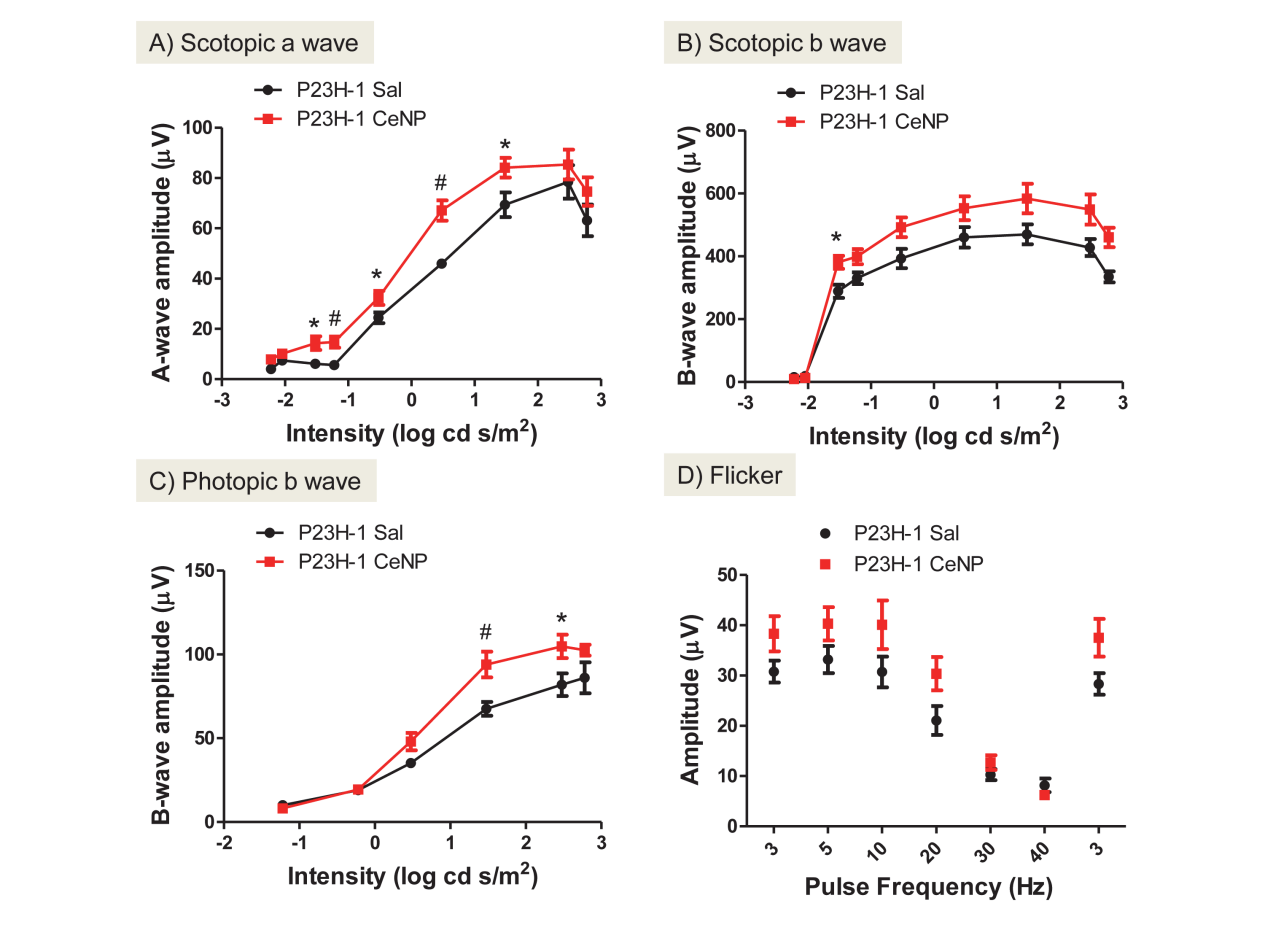
CeNPs protected rod and cone cell functions in P23H-1 rats after a single intravitreal injection (172 ng per eye) when evaluated 24 days post injection (dpi). The average increases for scotopic a-wave amplitudes was 180%, and 133% for photopic b-wave amplitudes across the intensities that showed statistically significant changes compared to saline-injected animals. *P<0.05, #P<0.01. N = 8–10 eyes from 4–5 animals per treatment group.

**Fig 2:**
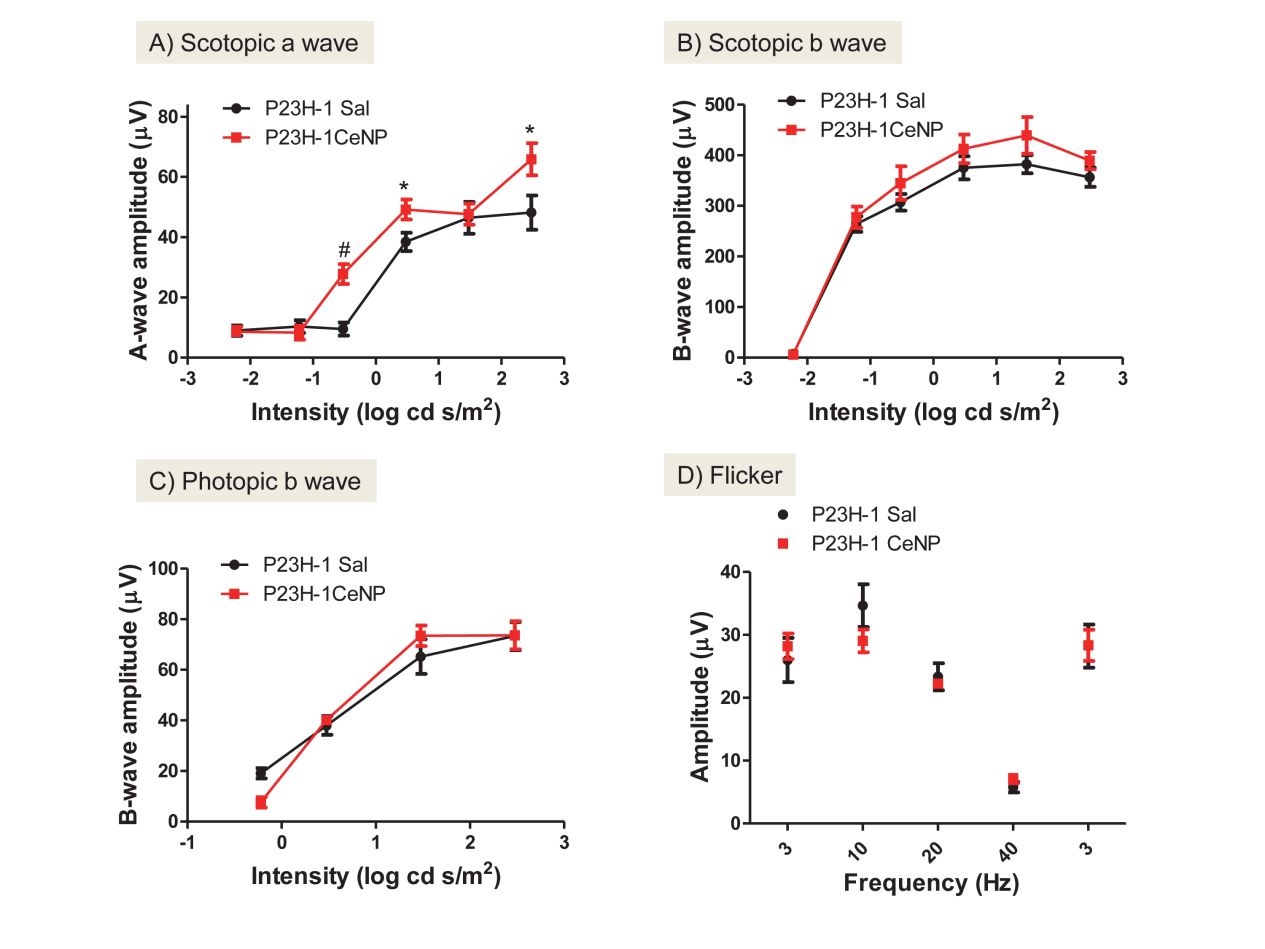
CeNPs protected rod cell function only in P23H-1 rats after a single intravitreal injection (344 ng per eye) when evaluated at 34 dpi. The average increase of scotopic a-wave amplitudes was 186% across the intensities that showed statistically significant changes compared to saline-injected animals. There was no change in the photopic b-wave amplitudes in the intensities tested, nor were there changes in the flicker ERG. *P<0.05, #P<0.01. N = 6–8 eyes from 3–4 animals per treatment group.

Measurements from electroretinogram (ERG) recordings enable us to evaluate the functions of rod and cone photoreceptor cells. Scotopic a-wave amplitude measures primarily the function of rod cells and scotopic b-wave amplitude the function of neurons that are post-synaptic to the rod cells, i.e. mostly bipolar cell function. Cone cell function is measured by photopic b-wave amplitude and flicker ERG after rod cells are temporarily inactivated [26]. When a single dose of CeNPs was injected intravitreally before 3 weeks of age, we observed increased rod cell function up to 34 days post injection (dpi; Figs 1 and 2). On average, we observed a 180% increase in a-wave amplitude over a wide range of flash intensities from -1.5 to 1.5 log cd s/m^2^ for measurements taken at 24 dpi. The amplitude increase at 34 dpi was observed at higher light intensities only. The increase was most dramatic at -0.5 log cd s/m^2^ (293%, Fig 2). The average increase of scotopic b-wave amplitudes was 123% for 24 dpi, and 113% for 34 dpi; these increases were not statistically significant. Dosages at 3.4 ng and 34.4 ng per eye were tested in the same experiment; we observed increased scotopic ERG amplitudes in the 344 ng group only when examined at 34 dpi (Fig 2 and data not shown). By 42 dpi or 6 weeks post injection, we did not observe changes in rod cell function between CeNPs- and saline-injected animals (Table 1 and data not shown). Interestingly, the cone cell protection offered by CeNPs had a shorter duration. We observed an average increase of 133% for photopic b-wave amplitude, and 132% for flicker amplitude in CeNPs injected animals in the 24 dpi group, but did not observe changes in amplitude in the 34 dpi group (Figs 1 and 2). Additionally, we also evaluated the effective minimum dosage of CeNPs ranging from 1.72 ng to 344 ng per eye using our functional assays (data not shown). We found that the minimum effective dose was 1μl of 1mM or 172 ng per eye. To reduce the effects of variability due to the delivery system, we routinely administer 344 ng or 2 μl of 1 mM CeNPs per eye.

**Table 1:** Effects of CeNPs on Rod and Cone Cell Functions of P23H-1 Rat after a Single Intravitreal Injection at Different Ages and Durations.

Expt #	Age at injection	Evaluation at dpi	Injection Volume	Scotopic a wave	Scotopic b wave	Photopic b wave	Flicker
1	P14	24	1 ul	Higher	Higher (NS)	Higher	Higher (NS)
2	P16	34	2 ul	Higher	Higher (NS)	No change	No change
3	P22	44	1 ul	No change	No change	No change	No change
4	P21	42	2 ul	No change	No change	No change	No change

ERG amplitudes were compared to saline injected ones. Abbreviations: dpi = days post injection, NS = not statistically significant

**Fig 3:**
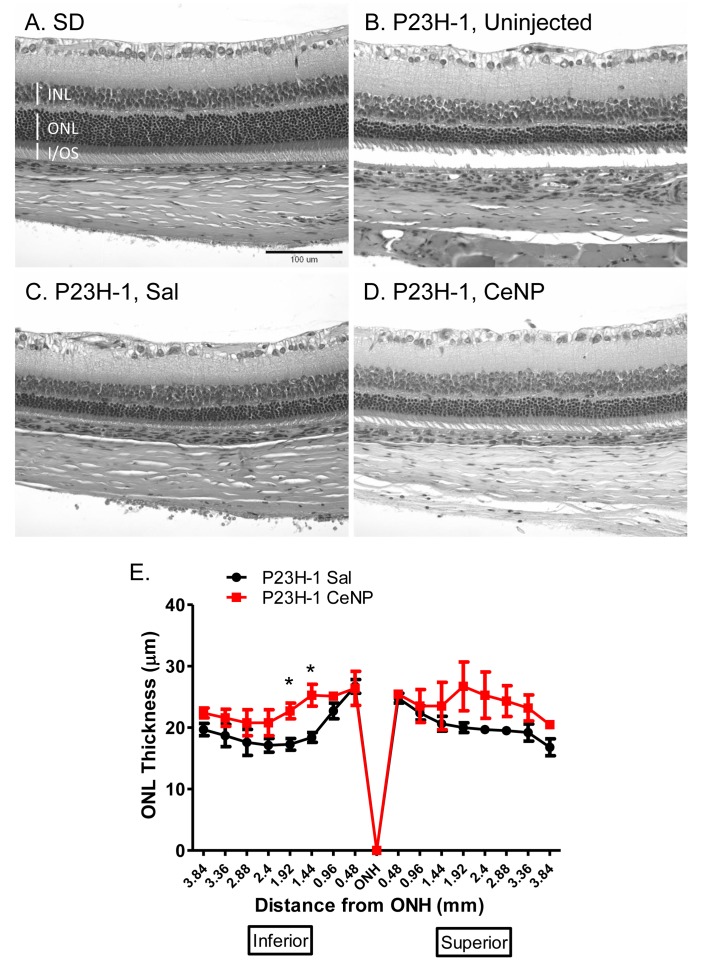
Morphometric analysis of ONL thickness in CeNPs- and saline- treated P23H-1 rats. One μl of 1 mM CeNPs (172 ng) or saline was delivered to each eye of the animals at P23 and eyes were harvested at 28 dpi; three eyes from 3 individuals were examined per treatment group. (A-D) Representative photomicrographs of H&E stained retinal sections from wildtype, Sprague Dawley (SD) or P23H-1 animals uninjected or treated with either saline or CeNPs. Similar regions were shown: 1 mm from the ONH in the inferior region. (E) shows quantification of the ONL thickness measurements. The overall ONL thickness was higher in CeNPs-treated than in saline-treated animals although the increases were not statistically significant across many of the regions. INL = inner nuclear layer, ONL = outer nuclear layer, I/OS = rod inner/outer segment, ONH = optic nerve head. *P<0.05.

The outer nuclear layer (ONL) contains the cell bodies of both rod and cone cells. Since 97% of the rodent photoreceptor cells are rods [27], the thickness of this layer correlates with the number of rod cells in the retina. We measured the ONL thickness of retinas obtained from animals injected at P25 and euthanized at 28 dpi. We saw an increase in ONL thickness of CeNPs-treated compared to saline-treated animals. However, the increase was not statistically significant (Fig 3). We quantified the amount of retinal proteins by Western blot analyses in animals treated with either saline or CeNPs at P16 and tissue collected at 27 dpi. We did not observe differences between the two treatment groups with regard to the protein levels of a rod-cell-specific protein, rod transducin-alpha (S1 Fig).

Because CeNPs were effective in delaying rod cell degeneration in the P23H-1 rats, we investigated the cellular mechanisms of CeNPs neuroprotection and their catalytic activities *in vivo* using this animal model. In subsequent experiments, we routinely delivered 344 ng (or 2 μl of 1 mM) of CeNPs in saline or 2 μl of saline alone to the vitreous of P15 pups when their eyelids were open.

### CeNPs Delayed Rod Cell Degeneration by Reduction of Apoptosis Shortly After Injection

**Fig 4:**
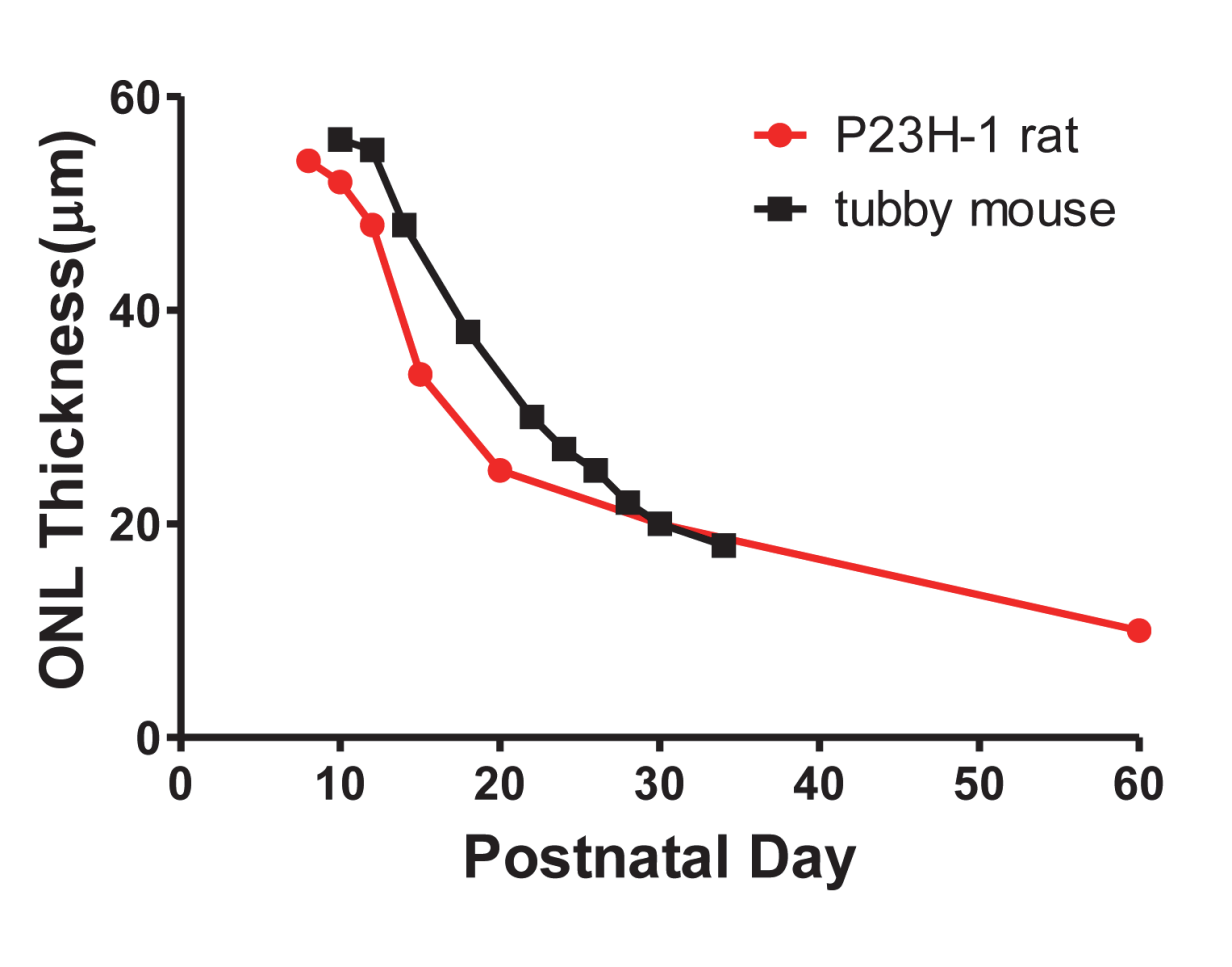
Rates of ONL thickness reduction in two photoreceptor degeneration rodent models: P23H-1 rat and *tubby* mouse. Data were adapted from [23] and [28] for P23H-1 rat and *tubby* mouse, respectively. The decline is most rapid between P12 and P20 for both models. In this direct comparison, P23H-1 rat has a more aggressive rod cell degeneration rate between P12 and P30 than the *tubby* mouse.

La Vail and colleagues showed that reduction of ONL thickness in the P23H-1 rat was fastest between P12 and P20 (Fig 4) [23]. Compared to another photoreceptor degeneration model, the *tubby* mouse [28], the P23H-1 rat has a more aggressive rod cell degeneration phenotype at these early stages. The death of rod cells slowed from P20 and beyond (Fig 4).

**Fig 5:**
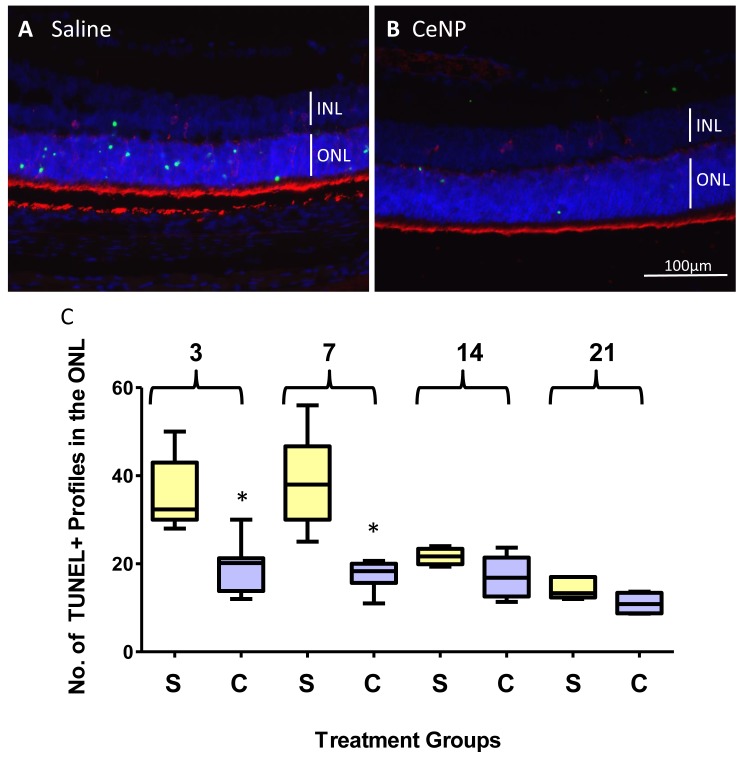
A single application of CeNPs at P15 reduced the number of apoptotic death of photoreceptor cells for at least 21 days. (A-B) Representative photomicrographs of retinal sections from 3 dpi group with examples of TUNEL+ profiles (green). Nuclei in the ONL and INL are labeled blue. Rod outer segments are labeled red with Rhodopsin, 1D4, antibody. CeNPs-treated animals have significantly fewer TUNEL+ profiles in the ONL compared to saline-treated ones. (C) Quantification of TUNEL+ profiles in the ONL from 3, 7, 14, and 21 dpi groups. The reductions from 3 and 7 dpi groups of CeNPs injected animals were highly significant. Statistical summaries from these box plots are detailed in Table 2. Abbreviations: dpi = days post injection, S = Saline, C = CeNPs, *p<0.05.

Irrespective of the genetic cause of photoreceptor cell degeneration, apoptosis is the common pathway that leads to cell death [29–31]. We hypothesized that CeNPs interrupted the cell death process by preventing apoptosis. We measured apoptosis in the ONL using the *in situ* terminal deoxyribonucleotidyl transferase (TDT)-mediated digoxigenin-dUTP nick-end labeling assay (TUNEL assay) which is a reliable method to detect cells *in situ* that are in the terminal phase of apoptosis [32,33]. After CeNPs were administered at P15, we observed a 46% reduction in apoptosis 3 dpi (Fig 5 and Table 2). The reduction was 56% at 7 dpi (Fig 5 and Table 2). By 14 and 21 dpi the reductions were at 21% and 24%, respectively (Fig 5 and Table 2).

**Table 2:** Statistical Summaries of TUNEL+ profiles in the ONL of Retinal Sections from CeNPs and Saline Treated P23H-1 Rats.

	3 dpi		7 dpi		14 dpi		21 dpi	
	Sal	CNP	Sal	CNP	Sal	CNP	Sal	CNP
Number of samples	7	8	7	7	4	6	5	4
Minimum	28	12	25	11	19.33	11.33	12	8.67
25% Percentile	30	13.83	30	15.67	19.92	12.58	12.33	8.753
Median	32.33	20.17	38	18.33	21.67	16.83	13.33	10.84
75% Percentile	43	21.25	46.67	20	23.42	21.42	17	13.42
Maximum	50	30	56	20.67	24	23.67	17	13.67
Mean	35.81	19.33	38.52	17.1	21.67	17.06	14.4	11
Std. Deviation	7.928	5.665	10.52	3.304	1.905	4.635	2.42	2.539
Std. Error	2.996	2.003	3.977	1.249	0.9526	1.892	1.082	1.27
P value oft test		0.0004*		0.0002*		0.1003		0.0797
% change relative to Sal	0%	-46%	0%	-56%	0%	-21%	0%	-24%
								*P<0.05

Animals were injected at P15 and eyes were harvested at 3, 7, 14, and 21 days post injection. Abbreviations: Sal = Saline, CNP = CeNP, dpi = days post injection.

### CeNPs Decreased Lipid Peroxidation in the Retinas of P23H-1 Rats

Products of lipid peroxidation, especially isoprostanes, are excellent markers for oxidative damage in biological samples. Isoprostanes are products of free radical-catalyzed peroxidation of arachidonic acid and are not generated by cyclooxygenase [34–36]. In comparison to other oxidative stress markers, isoprostanes are more accurate and sensitive [37]. These end products are chemically stable and are long lasting markers for assessment of oxidant injury.

**Fig 6:**
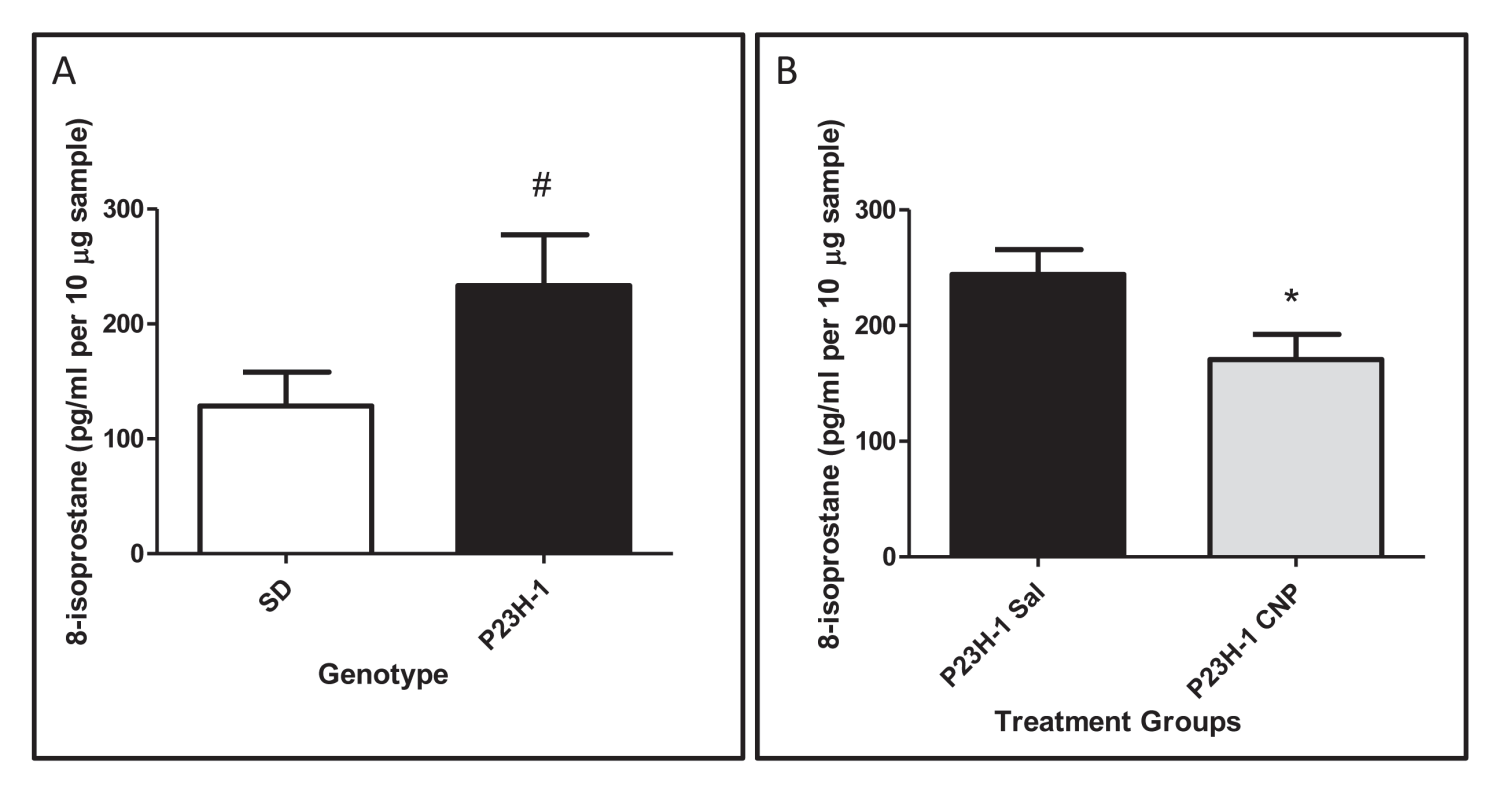
Quantification of lipid peroxidation in retinal samples of P23H-1 rats. (A) Retinas of 28-day old P23H-1 rats had elevated levels of 8-isoprostanes compared to age-matched wildtype, SD controls. N = 4, #P<0.01. (B) A single CeNPs application at P15 reduced the level of 8-isoprostanes 14 days later in the retinas of P23H-1 rats. N = 5–6, *P<0.05. Abbreviations: SD = Sprague Dawley, Sal = Saline, CNP = CeNPs.

Unlike other lipid hydroperoxide assays that provide a snapshot view of the level of lipid peroxidation at the time of assay, tissue levels of 8-isoprostane reflects the integrated level of lipid peroxidation over time. We quantified the total amount of 8-isoprostane in retinal samples by hydrolyzing the esterified fatty acid into free 8-isoprostanes. We observed a marked increase (180%) in 8-isoprostane concentration in retinal tissues from P23H-1 rats compared to wildtype, SD animals at P28 (Fig 6A). When a single dose of CeNPs (344 ng) was administered at P15, we observed a 30% reduction of 8-isoprostane in the retinas of the transgenic animals compared to saline injected ones 14 dpi (Fig 6B). These data represent the first quantitative assessment of oxidative stress in a rodent retinal disease model.

## Discussion

### A Single Application of CeNPs Slowed the Functional Decline of Rod and Cone Cells in P23H-1 Rats

After evaluating a range of dosages (1.72 to 344 ng) and periods of effectiveness (up to 44 dpi) of CeNP treatment, we demonstrated that when at least 172 ng of CeNPs were applied intravitreally before or around 3-week of age, both rod (on average ~180% of saline-treated) and cone (on average ~133% of saline-treated) functions of P23H-1 rats were enhanced 24 dpi. At 34 dpi we observed enhanced rod (~186% of saline-treated), but not cone function at 344 ng CeNP dosage. The duration of enhanced photoreceptor cell function after CeNPs treatment is shorter in this rat model than is observed in the *tubby* mouse photoreceptor degeneration model [9] which also exhibits similar rapid early rod cell loss as the P23H-1 rats (Fig 4). In the *tubby* experiment, 172 ng of CeNPs were intravitreally delivered to *tubby* newborn mice at P7. We [9] observed enhanced mixed rod and cone cell functions (~150% compared to saline-injected) up to 73 dpi. In those experiments, one single strong intensity flash (600 cd s/m^2^ or 2.78 log cd s/m^2^) was employed. Interestingly, we also observed a shorter neuroprotective period for cone cell function in the *tubby* model. The enhanced cone function (~160% of saline-injected) recorded at 1000 cd s/m^2^ or 3 log cd s/m^2^ was observed up to 42 dpi only, 31 days shorter than the observation for rod cell function. We speculate that the shorter duration for the enhanced photoreceptor cell function provided by CeNPs in the rat model may be due to 1) a later application time point of CeNPs, and 2) the relative dosage of CeNPs in the rat eye compared to the mouse eye. In this set of experiments, we delivered CeNPs at P15 or later when the eyelids of the animals were open vs in the *tubby* mouse experiments at P7 when the eyelids stayed closed for another 7 days after injection. We speculate that the earlier the reduction of cellular oxidative stress, the longer the cells can stay functional due to reduced oxidative damage. Additionally, we think that the dosage applied significantly contributes to the differences observed. The volume of an adult mouse vitreous is 5.3 μl vs 54.4 μl in the adult rat. The retinal area of an adult mouse is 15.6 mm^2^ vs 52 mm^2^ in the adult rat [38]. Assuming 94% of the injected CeNPs reached the retina [16], the dosage would be 10.4 ng/ mm^2^ in the mouse and 6.2 ng/ mm^2^ in the rat retina. We conclude that the available neuroprotective data from two similarly rapid photoreceptor degeneration models but of different eye sizes, equip us to better design pre-clinical and clinical trial studies of CeNPs for larger animals and humans. The retinal surface of a human eye is ~1024 mm^2^ [38]; the lowest effective dosage would be 6754 ng or 39 μl of 1 mM CeNPs. Relative to the vitreal volume of 5200 μl in human [38], 39 μl can be readily delivered without concern for cellular damage due to elevation of intraocular pressure [39].

We notice that changes from the morphometric studies of ONL thickness were not statistically significant; we speculate that the methodology may not be sensitive enough to detect the difference and/or the sample size may be too small. This result paralleled our observation of our Western blot data where we failed to detect statistically significant difference of rod retinal proteins between CeNPs and saline injected animals when assayed at 27 dpi (S1C and S1D Fig, data not shown). We postulate that the neuroprotective effect may be more apparent if we had performed the assay at an earlier time point, for example, at 14 dpi, and we found that this was indeed the case (S1A and S1B Fig). Finally, we think that the functional assay is more sensitive and robust in assessing the overall health of the light sensing portion of the neuroretina because functional assays evaluate the health status of the entire photoreceptor cells and their connections to other cell types not just the presence/absence of photoreceptor cell bodies in the ONL.

### CeNPs Application Reduced the Cell Death Index of Photoreceptor Cells in P23H-1 Rats in a Time-Dependent Fashion

The process of apoptosis consists of three phases: initiation, commitment, and degradation. The estimated duration from initiation to degradation ranges from 6 to 24 hours with the greatest variation in the initiation phase [40]. The TUNEL assay detects cells that are at the degradation phase which is estimated to be about 5 hours [41]. The clearance of TUNEL positive profiles in the developing rat cerebral cortex is about 3 hours [42]. Because apoptosis is a conserved cell death process [43], we think that these parameters will apply to the rod cells in the P23H-1 rats. Monitoring apoptosis by *in situ* TUNEL assay provides a temporal snapshot of the health status of cells in the tissue. Understandably, cells that have not begun the degradation phase and those that have completed the degradation phase will be missed using this method and likely causes an undercount of the number of apoptotic cells. Nonetheless, the cell death index by TUNEL assay is useful to assess the therapeutic activities or pharmacodynamics of therapeutic agents because TUNEL+ profiles detected at each time point greater than 5 hours apart would be from cells that have not been counted previously. We demonstrated that CeNPs were effective in reducing apoptotic photoreceptor cells up to 21 days after intravitreal injection. The anti-apoptotic effect was most striking up to 7 days after CeNPs application when we observed 56% reduction of TUNEL+ profiles in the ONL. The data represent the numbers of TUNEL+ profiles in single 5 μm thick retinal sections through the central part of the eye. If we extrapolate that number to the whole retina, the number of dying cells will be in the thousands every day. For example, the estimated TUNEL+ profiles in the whole retina at P22 would be ~62,000 at the time of tissue harvesting. If we sample the retina every six hours, we would observe a similar number from P18 to P22. We estimate that about a quarter of a million rod cells are dying every day. In five days, 1.25 million rod cells would have died and this is a gross underestimate of the dead rod cells because by P28, approximately half of the rod cells present at P15 would have disappeared! (There are about 22 million rod cells in the adult rat retina. Data extrapolated from [38] and [27].)

Surprisingly, a single application of CeNPs at P15 reduced the number of TUNEL+ profiles by half for up to 7 dpi. Because a large percentage of rod cells in the P23H-1 rat retina are destined to die early and fast, we hypothesize that CeNPs are delaying the initiation of the apoptotic death of these rod cells. Because CeNPs possess catalytic ROS scavenging activities, we hypothesize that CeNPs are able to maintain the redox balance of these “sick” cells for a longer period. The catalytic activity of CeNPs continued for the next 14 days, albeit at a much reduced level. We observed small (~20%) although not statistically significant reduction even at 21 dpi. From this study, we conclude that the pharmacodynamics of CeNPs is maximal between 3 and 7 days post injection in the P23H-1 rats at the dosage applied and the catalytic activity of CeNPs becomes limiting at 14 dpi. An alternative, but not mutually exclusive, interpretation is that the smaller reduction in cell death index at the later time points reflects a different cause of rod cell death that cannot be alleviated by the ROS scavenging activity of CeNPs. Furthermore, to determine if the dose of CeNPs is limiting, we can increase the initial dosage or apply a second dose 7 days after the first dose and perform the TUNEL assay at equivalent time intervals.

Even though we have demonstrated that 94% of injected CeNPs are taken up by retinal cells within one hour of intravitreal injection [16], it is still unknown how quickly cellular effects can be detected after CeNPs application *in vivo*. In primary rat retinal cell cultures, the earliest effect of CeNPs mediated reduction of ROS induced by hydrogen peroxide treatment was found to be at 12 hours post incubation and not at 30 min [25]. Using this cell death index paradigm, we are confident that one can determine when CeNPs start to exert their catalytic activity after intravitreal injection.

### P23H-1 Rat Retinas Had Elevated Oxidative Stress and CeNPs Reduced it

We showed that the retinas of P23H-1 rats had significantly higher levels of 8-isoprostane (80% higher) at 4-week of age than wildtype, SD animals of the same age. Because 8-isoprostane is a stable end product of lipid peroxidation, the measured level reflects the accumulation of this end product up to the point of tissue harvesting. Even though by this age (P28) the ONL thickness of P23H-1 rats is only 50% of the wildtype (20 μm vs 40 μm) [23], the amount of lipid peroxidation product is 80% higher in the remaining amount of the retina. Approximately 76% of total rodent retinal neurons are represented by rod cells [27]. We estimate that the remainder cells have nearly 3 times (2.9 X) as much 8-isoprostanes as in wildtype retinal cells. Our data clearly demonstrate that oxidative stress is increased in this adRP model. This finding is consistent with other studies that show increased oxidative stress in neurodegenerative diseases and our data are the first to provide a quantitative assessment of the oxidative damage.

We showed that a single injection of 344 ng of CeNPs on P15 was able to reduce 8-isoprostane by 30% 14 dpi in the retinas of P23H-1 rats. We postulate that the reduction likely commenced shortly after CeNPs administration and persisted up to the point of tissue harvesting. This observation is consistent with two interpretations: 1) CeNPs lower oxidative damage in the P23H-1 retinas by reducing ROS level, and/or 2) CeNPs reduce the number of apoptotic cells and as a consequence lower the oxidative stress marker. Recognizing the unique physical and chemical properties of CeNPs, we favor the first interpretation of which CeNPs scavenge ROS to lower oxidative stress inside cells and thus prolonging the life span of post-mitotic rod photoreceptor cells.

## Materials and Methods

### 1) Animals

We obtained a breeding colony of P23H line 1 (P23H-1) homozygous rats from Dr Matt LaVail from the University of California, San Francisco [23]. We have a breeding colony of Sprague-Dawley (SD) albino rats; offspring from these were used as age-matched wildtype controls. We crossed homozygous P23H-1 males to SD females to obtain heterozygous P23H-1 F1 offspring for experiments. All animals were housed in the Dean McGee Eye Institute (DMEI) vivarium and were kept under cyclic light conditions (12 h on/12 h off, 5–20 lux).

### 2) Ethics Statement

Animals were cared for and handled according to the Association for Research in Vision and Ophthalmology statement for the use of animals in vision and ophthalmic research. The study was approved by the University of Oklahoma Health Sciences Center Institutional Animal Care and Use Committee (OUHSC IACUC) and the DMEI IACUC. The approved protocol numbers were 11–089 from OUHSC IACUC, and D-11-089 from the DMEI IACUC.

### 3) Synthesis of CeNPs

CeNPs were synthesized using simple wet chemistry methods as described previously [44]. Extensive detailed characterization results of as synthesized CeNPs can be found in the supplemental materials (Text S1 and Fig. S1) in [8]. Our stable water-dispersed CeNPs were 3–5 nm in size. The size of these particles remained the same in a wide range of pH buffers and upon aging [45]. Our synthesized CeNPs show uniform round morphology using high resolution transmission electron microscopy imaging. The surface area was determined using BET method and found to be 91 m^2^/g. These particles do not elicit toxic effects in the healthy rat retinas [16].

### 4) Intravitreal injection of CeNPs

Postnatal day (P) 15 or P21 P23H-1 rats were selected for intravitreal injection. Animals were anesthetized by intraperitoneal injection of a mixture of ketamine (80 mg/kg) and xylazine (4mg/kg). Pupils were dilated by application of a drop of phenylephrine (10% solution) to the cornea before the delivery of 1 or 2 μl of 1 mM CeNPs in saline (i.e. 172 or 344 ng, respectively) or 1 or 2 μl saline only into the vitreous with the aid of an ophthalmic operating microscope. Both eyes of each animal received the same treatment.

### 5) Electroretinogram Recordings (ERG)

Detailed description of the methods for scotopic, photopic and flicker ERG can be found in [16]. Scotopic a-wave amplitude reflects primarily the function of rod cells. Scotopic b-wave amplitude reflects the function of neurons in the inner retina predominately bipolar neurons which are post-synaptic to rod photoreceptor cells. Photopic b-wave amplitude and flicker ERG reflect the function of cone cells [26]. Each data point represented the average from at least three individual rats or six eyes.

### 6) Measuring outer nuclear layer (ONL) thickness of retinal sections

P23H-1 heterozygous animals were injected with 1 μl of either saline or CeNPs (172 ng) on P23. Eyes were harvested 28 days post injection following procedures described in [6]. They were fixed in Perfix and embedded in paraffin. Five μm thick retinal sections were obtained along the superior and inferior axis through the optic nerve head (ONH). Hematoxylin-eosin stained sections were viewed under a 20X objective of a Nikon E400 microscope. ONL thickness was measured from the ONH to the ora serrata in both directions at 480 μm intervals. Each data point represented the average measurements of 3 eyes from 3 different animals.

### 7) Western blot analysis

After eye enucleation, the retina was dissected from the eye cup. Two hundred μl of homogenization buffer (62.5 mM Tris-HCl, pH 6.8; containing protease and phosphatase inhibitors; Roche cat# 11873580 and 04906837001, respectively) was added. Tissue disruption and homogenization was achieved by using the steel bead method with a TissueLyzer. Using a 7 mm steel bead/sample, we set the TissueLyzer LT (Qiagen) at 50 Hz for 2 minutes. Samples were centrifuged at 13000 rpm at 4°C for 20 minutes. Supernatant from each sample was collected in a new tube, and protein concentration was determined using the BCA protein assay kit (Thermo Scientific, cat. # 23227). The pellet portion (membrane proteins) was resuspended in 200 μl of homogenization buffer and the TissueLyzer procedure was repeated again.

We used 20 μg of proteins per sample to run 10% SDS-PAGE. After electrophoresis, proteins were transferred to 0.45 μm pore-size nitrocellulose membrane (Bio-Rad) using the Mini Trans-Blot Cell (Bio-Rad, cat. # 170–3930). Immunolabeling of nitrocellulose membrane was performed according to [8]. Antibodies (ab) used were: rod specific markers: 1D4 (1:20000; rhodopsin ab, gift from Robert Molday [46]), rod transducin-alpha ab (1:2000; Santa Cruz Biotechnologies cat# SC389); rod arrestin ab, MH785 (1:1000; a kind gift from P. Hargrave, University of FL at Gainsville). Secondary ab’s conjugated to HRP (1:5000) were from Jackson ImmunoResearch. We used the SuperSignal West Dura Substrate (Thermo Scientific cat. # 34076) for antibody detection. Western blot images were obtained using a Kodak 4000R Image Station. We quantified the relative expression level by measuring the net intensity of the appropriate band size normalized to the level of GAPDH using the Kodak Molecular Imaging Software v 4.0.5. Three to four eyes were used for each treatment group. Depending on the experiment, eyes could be from different animals or from the same animal receiving the same treatment in both eyes.

### 8) TdT-mediated digoxigenin-dUTP nick-end labeling assay (TUNEL assay)

Five μm thick paraffin retinal sections through the optic nerve head (ONH) were used for TUNEL assay. Eyes were harvested as described in [16]. We quantified the number of apoptotic (TUNEL+) profiles in the outer nuclear layer (ONL) of the retinas using the ApopTag Plus Fluorescein In Situ Apoptosis Detection kit (Millipore Cat. #S7111). We ensured that analyzed sections were all from the central part of the eye. Measurements were averaged from three consecutive retinal sections. Intensity of green fluorescently labeled objects was evaluated using an arbitrary scale of 1–3 (low, med, high) and we counted those which were scored as “3”. We counted labeled-objects as TUNEL+ profiles if their diameters were 2.4 μm or above in both length and width and if they were in the ONL or outer plexiform layer (OPL) only. The majority of TUNEL+ profiles were found in the ONL. Samples were analyzed using a Nikon E800 microscope with a 40X objective. Images were captured using a CCD MicroMAX camera (Princeton Instruments) with the MetaVue software (Molecular Devices). Photomicrographs of TUNEL-labeled retinal sections are shown in Fig 5A and 5B. Sample sizes for each treatment group at each time point are indicated in Table 2.

### 9) 8-isoprostane assay

Quantitative measurement of lipid oxidative damage in the retina was assessed by measuring free 8-isoprostane using the 8-isoprostane EIA kit (Cayman Chemical Company Cat. # 516360). The 8-isoprostane EIA kit assay measures the cumulative lipid peroxidation product of 8-isoprostane in tissue samples. We targeted lipid peroxidation by-products as markers for oxidative damage because the retina is enriched in polyunsaturated fatty acids (PUFA) [47]. PUFAs are more vulnerable to oxidation in the high metabolic environment of the retina. Retinas were harvested as described above and tissue disruption and homogenization were achieved the same way as described for Western blot analysis, except that tissue was resuspended in 0.1 M potassium phosphate buffer, pH 7.4, 1mM EDTA and 0.005% butylated hydroxytoluene, according to the product handbook. Supernatant was collected after centrifugation (8000g for 10 minutes) and hydrolyzed to obtain free 8-isoprostane. Protein concentration was determined using the BCA protein assay kit (Thermo Scientific, cat. # 23227) for sample normalization. Unlike many lipid hydroperoxide assay kits that provide a snapshot view of level of lipid peroxidation at the time of assay, tissue levels of 8-isoprostane reflects the integrated level of lipid peroxidation over time. Sample sizes are indicated in the figure legend of Fig 6.

### 10) Statistical analysis

Values were expressed as means ± SEM. Statistical analyses were performed using student t-test using GraphPad Prism version 5.00 for Windows (GraphPad Software, San Diego CA USA, www.graphpad.com). A P value less than 0.05 was considered significant and is indicated by an asterisk.

## Supporting Information

S1 Fig Rod transducin alpha (rTa) expression level in retinal extracts of P23H-1 rats. Animals were injected with either 344 ng CeNPs in 2 μl of saline or saline alone on P16. Eyes were harvested 14 days post injection (dpi) (A, B) or 27 dpi (C, D). We detected significantly higher levels of rTa in CeNPs treated rats at 14 dpi but not at 27 dpi. Additionally, we observed similar increase in arrestin and rhodopsin protein expressions in retinal extracts from P16 injected animals and retinas harvested 14 dpi (data not shown). We performed student t-test to compare the mean values (± std error of mean) of the two treatment groups. *P<0.05.
(PDF)

## References

[1] Andersen JK. Oxidative stress in neurodegeneration: cause or consequence? Nature medicine. 2004;10 Suppl:S18–25. 15298006 10.1038/nrn1434

[2] Fatokun AA, Stone TW, Smith RA. Oxidative stress in neurodegeneration and available means of protection. Front Biosci. 2008;13:3288–311. 18508433 10.2741/2926

[3] Kowluru RA, Chan PS. Oxidative stress and diabetic retinopathy. Exp Diabetes Res. 2007;2007:43603 17641741 10.1155/2007/43603 PMC1880867

[4] Hollyfield JG, Bonilha VL, Rayborn ME, Yang X, Shadrach KG, Lu L, et al Oxidative damage-induced inflammation initiates age-related macular degeneration. Nat Med. 2008;14(2):194–8. 10.1038/nm1709 18223656 PMC2748836

[5] Aslan M, Cort A, Yucel I. Oxidative and nitrative stress markers in glaucoma. Free Radic Biol Med. 2008;45(4):367–76. 10.1016/j.freeradbiomed.2008.04.026 18489911

[6] Chen J, Patil S, Seal S, McGinnis JF. Rare earth nanoparticles prevent retinal degeneration induced by intracellular peroxides. Nat Nanotechnol. 2006;1(2):142–50. 10.1038/nnano.2006.91 18654167

[7] Kong L, Cai X, Zhou X, Wong LL, Karakoti AS, Seal S, et al Nanoceria extend photoreceptor cell lifespan in tubby mice by modulation of apoptosis/survival signaling pathways. Neurobiology of disease. 2011;42(3):514–23. 10.1016/j.nbd.2011.03.004 21396448 PMC3411120

[8] Zhou X, Wong LL, Karakoti AS, Seal S, McGinnis JF. Nanoceria inhibit the development and promote the regression of pathologic retinal neovascularization in the Vldlr knockout mouse. PLoS ONE. 2011;6(2):e16733 10.1371/journal.pone.0016733 21364932 PMC3043063

[9] Cai X, Sezate SA, Seal S, McGinnis JF. Sustained protection against photoreceptor degeneration in tubby mice by intravitreal injection of nanoceria. Biomaterials. 2012;33(34):8771–81. 10.1016/j.biomaterials.2012.08.030 22959465 PMC4176881

[10] Kyosseva SV, Chen L, Seal S, McGinnis JF. Nanoceria inhibit expression of genes associated with inflammation and angiogenesis in the retina of Vldlr null mice. Exp Eye Res. 2013;116:63–74. 10.1016/j.exer.2013.08.003 23978600 PMC4263290

[11] Cai X, Seal S, McGinnis JF. Sustained inhibition of neovascularization in vldlr-/- mice following intravitreal injection of cerium oxide nanoparticles and the role of the ASK1-P38/JNK-NF-kappaB pathway. Biomaterials. 2014;35(1):249–58. 10.1016/j.biomaterials.2013.10.022 24140045 PMC3911773

[12] Korsvik C, Patil S, Seal S, Self WT. Superoxide dismutase mimetic properties exhibited by vacancy engineered ceria nanoparticles. Chem Commun (Camb). 2007(10):1056–8. 17325804 10.1039/b615134e

[13] Pirmohamed T, Dowding JM, Singh S, Wasserman B, Heckert E, Karakoti AS, et al Nanoceria exhibit redox state-dependent catalase mimetic activity. Chem Commun (Camb). 2010;46(16):2736–8. 10.1039/b922024k 20369166 PMC3038687

[14] Das M, Patil S, Bhargava N, Kang JF, Riedel LM, Seal S, et al Auto-catalytic ceria nanoparticles offer neuroprotection to adult rat spinal cord neurons. Biomaterials. 2007;28(10):1918–25. 17222903 10.1016/j.biomaterials.2006.11.036 PMC1913191

[15] Celardo I, Traversa E, Ghibelli L. Cerium oxide nanoparticles: a promise for applications in therapy. J Exp Ther Oncol. 2011;9(1):47–51. 21275265

[16] Wong LL, Hirst SM, Pye QN, Reilly CM, Seal S, McGinnis JF. Catalytic nanoceria are preferentially retained in the rat retina and are not cytotoxic after intravitreal injection. PLoS ONE. 2013;8(3):e58431 10.1371/journal.pone.0058431 23536794 PMC3594235

[17] Ferrari S, Di Iorio E, Barbaro V, Ponzin D, Sorrentino FS, Parmeggiani F. Retinitis pigmentosa: genes and disease mechanisms. Current genomics. 2011;12(4):238–49. 10.2174/138920211795860107 22131869 PMC3131731

[18] Farrar GJ, Millington-Ward S, Chadderton N, Humphries P, Kenna PF. Gene-based therapies for dominantly inherited retinopathies. Gene therapy. 2012;19(2):137–44. 10.1038/gt.2011.172 22089493

[19] Gorbatyuk MS, Knox T, LaVail MM, Gorbatyuk OS, Noorwez SM, Hauswirth WW, et al Restoration of visual function in P23H rhodopsin transgenic rats by gene delivery of BiP/Grp78. Proceedings of the National Academy of Sciences of the United States of America. 2010;107(13):5961–6. 10.1073/pnas.0911991107 20231467 PMC2851865

[20] Hartong DT, Berson EL, Dryja TP. Retinitis pigmentosa. Lancet. 2006;368(9549):1795–809. 17113430 10.1016/S0140-6736(06)69740-7

[21] Dryja TP, McGee TL, Reichel E, Hahn LB, Cowley GS, Yandell DW, et al A point mutation of the rhodopsin gene in one form of retinitis pigmentosa. Nature. 1990;343(6256):364–6. 2137202 10.1038/343364a0

[22] Lewin AS, Drenser KA, Hauswirth WW, Nishikawa S, Yasumura D, Flannery JG, et al Ribozyme rescue of photoreceptor cells in a transgenic rat model of autosomal dominant retinitis pigmentosa. Nat Med. 1998;4(8):967–71. 9701253 10.1038/nm0898-967

[23] La Vail MM. Retinal Degeneration Rat Model Resource. 2005. Available from: http://www.ucsfeye.net/mlavailRDratmodels.shtml. Accessed April 27, 2012.

[24] Machida S, Kondo M, Jamison JA, Khan NW, Kononen LT, Sugawara T, et al P23H rhodopsin transgenic rat: correlation of retinal function with histopathology. Invest Ophthalmol Vis Sci. 2000;41(10):3200–9. 10967084

[25] Chen J, Patil S, Seal S, McGinnis JF. Nanoceria particles prevent ROI-induced blindness. Adv Exp Med Biol. 2008;613:53–9. 10.1007/978-0-387-74904-4_5 18188928

[26] Perlman I. The Electroretinogram: ERG [online]. Salt Lake City, Utah, USA: University of Utah 2011 [updated 3/30/11. Online Textbook of the Visual System]. Available from: http://webvision.med.utah.edu/book/electrophysiology/the-electroretinogram-erg/. Accessed 23 February 2015.

[27] Jeon CJ, Strettoi E, Masland RH. The major cell populations of the mouse retina. The Journal of neuroscience: the official journal of the Society for Neuroscience. 1998;18(21):8936–46. 9786999 10.1523/JNEUROSCI.18-21-08936.1998 PMC6793518

[28] Kong L, Li F, Soleman CE, Li S, Elias RV, Zhou X, et al Bright cyclic light accelerates photoreceptor cell degeneration in tubby mice. Neurobiology of disease. 2006;21(3):468–77. 16216520 10.1016/j.nbd.2005.08.017

[29] Chang GQ, Hao Y, Wong F. Apoptosis: final common pathway of photoreceptor death in rd, rds, and rhodopsin mutant mice. Neuron. 1993;11(4):595–605. 8398150 10.1016/0896-6273(93)90072-y

[30] Portera-Cailliau C, Sung CH, Nathans J, Adler R. Apoptotic photoreceptor cell death in mouse models of retinitis pigmentosa. Proceedings of the National Academy of Sciences of the United States of America. 1994;91(3):974–8. 8302876 10.1073/pnas.91.3.974 PMC521436

[31] Marigo V. Programmed cell death in retinal degeneration: targeting apoptosis in photoreceptors as potential therapy for retinal degeneration. Cell Cycle. 2007;6(6):652–5. 17374995 10.4161/cc.6.6.4029

[32] Gavrieli Y, Sherman Y, Ben-Sasson SA. Identification of programmed cell death in situ via specific labeling of nuclear DNA fragmentation. The Journal of cell biology. 1992;119(3):493–501. 1400587 10.1083/jcb.119.3.493 PMC2289665

[33] Gold R, Schmied M, Giegerich G, Breitschopf H, Hartung HP, Toyka KV, et al Differentiation between cellular apoptosis and necrosis by the combined use of in situ tailing and nick translation techniques. Laboratory investigation; a journal of technical methods and pathology. 1994;71(2):219–25. 8078301

[34] Morrow JD, Hill KE, Burk RF, Nammour TM, Badr KF, Roberts LJ 2nd. A series of prostaglandin F2-like compounds are produced in vivo in humans by a non-cyclooxygenase, free radical-catalyzed mechanism. Proceedings of the National Academy of Sciences of the United States of America. 1990;87(23):9383–7. 2123555 10.1073/pnas.87.23.9383 PMC55169

[35] Milne GL, Yin H, Morrow JD. Human biochemistry of the isoprostane pathway. The Journal of biological chemistry. 2008;283(23):15533–7. 10.1074/jbc.R700047200 18285331 PMC2414280

[36] Roberts LJ 2nd, Milne GL. Isoprostanes. J Lipid Res. 2009;50 Suppl:S219–23. 10.1194/jlr.R800037-JLR200 18957694 PMC2674741

[37] Kadiiska MB, Gladen BC, Baird DD, Germolec D, Graham LB, Parker CE, et al Biomarkers of oxidative stress study II: are oxidation products of lipids, proteins, and DNA markers of CCl4 poisoning? Free radical biology & medicine. 2005;38(6):698–710. 15721980 10.1016/j.freeradbiomed.2004.09.017

[38] Marc RE. Rodent and Human Eye measurements. Available from: http://prometheus.med.utah.edu/~marclab/eyes.pdf. Accessed 15 April 2014.

[39] Falkenstein IA, Cheng L, Freeman WR. Changes of intraocular pressure after intravitreal injection of bevacizumab (avastin). Retina. 2007;27(8):1044–7. 18040242 10.1097/IAE.0b013e3180592ba6

[40] Saraste A, Pulkki K. Morphologic and biochemical hallmarks of apoptosis. Cardiovascular research. 2000;45(3):528–37. 10728374 10.1016/s0008-6363(99)00384-3

[41] Goldstein JC, Kluck RM, Green DR. A single cell analysis of apoptosis. Ordering the apoptotic phenotype. Annals of the New York Academy of Sciences. 2000;926:132–41. 11193030 10.1111/j.1749-6632.2000.tb05607.x

[42] Thomaidou D, Mione MC, Cavanagh JF, Parnavelas JG. Apoptosis and its relation to the cell cycle in the developing cerebral cortex. The Journal of neuroscience: the official journal of the Society for Neuroscience. 1997;17(3):1075–85. 8994062 10.1523/JNEUROSCI.17-03-01075.1997 PMC6573180

[43] Inohara N, Nunez G. Genes with homology to mammalian apoptosis regulators identified in zebrafish. Cell death and differentiation. 2000;7(5):509–10. 10917738 10.1038/sj.cdd.4400679

[44] Karakoti AS, Monteiro-Riviere NA, Aggarwal R, Davis JP, Narayan RJ, Self WT, et al Nanoceria as Antioxidant: Synthesis and Biomedical Applications. JOM (1989). 2008;60(3):33–7. 20617106 10.1007/s11837-008-0029-8 PMC2898180

[45] Vincent A, Inerbaev TM, Babu S, Karakoti AS, Self WT, Masunov AE, et al Tuning hydrated nanoceria surfaces: experimental/theoretical investigations of ion exchange and implications in organic and inorganic interactions. Langmuir: the ACS journal of surfaces and colloids. 2010;26(10):7188–98. 10.1021/la904285g 20131920 PMC2876981

[46] MacKenzie D, Molday RS. Organization of rhodopsin and a high molecular weight glycoprotein in rod photoreceptor disc membranes using monoclonal antibodies. Journal of Biological Chemistry. 1982;257(12):7100–5. 7085619

[47] Fliesler SJ, Anderson RE. Chemistry and metabolism of lipids in the vertebrate retina. Prog Lipid Res. 1983;22(2):79–131. 6348799 10.1016/0163-7827(83)90004-8

