# Supplementary material for: Defining the Catalytic Activity of Nanoceria in the P23H-1 Rat, a Photoreceptor Degeneration Model
Source: PLoS One. 2015 Mar 30;10(3):e0121977. doi: 10.1371/journal.pone.0121977 (PMC4379093; doi:10.1371/journal.pone.0121977)
Supplement: S1 Fig — Animals were injected with either 344 ng CeNPs in 2 μl of saline or saline alone on P16. Eyes were harvested 14 days post injection (dpi) (A, B) or 27 dpi (C, D). We detected significantly higher levels of rTa in CeNPs treated rats at 14 dpi but not at 27 dpi. Additionally, we observed similar increase in arrestin and rhodopsin protein expressions in retinal extracts from P16 injected animals and retinas harvested 14 dpi (data not shown). We performed student t-test to compare the mean values (± std error of mean) of the two treatment groups. *P<0.05. (PDF) [file pone.0121977.s001.pdf]

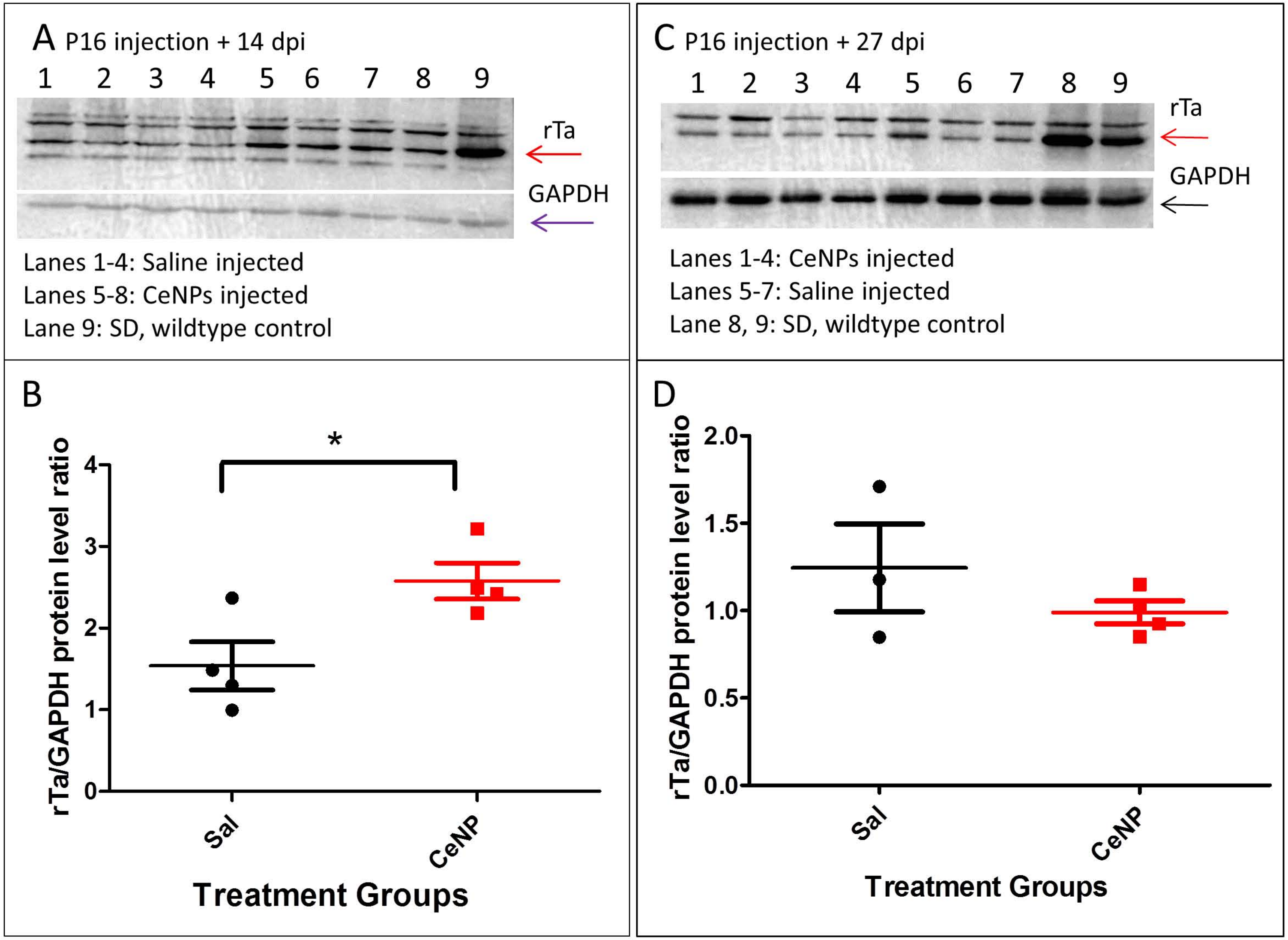

S1 Figure. Rod transducin alpha (rTa) expression level in retinal extracts of P23H-1 rats. Animals were injected with either 344 ng CeNPs in 2 ul of saline or saline alone on P16. Eyes were harvested 14 days post injection (dpi) (A, B) or 27 dpi (C, D). We detected significantly higher levels of rTa in CeNPs treated rats at 14 dpi but not at 27 dpi. Additionally, we observed similar increase in arrestin and rhodopsin protein expressions in retinal extracts from P16 injected animals and retinas harvested 14 dpi (data not shown). We performed student t-test to compare the mean values (± std error of mean) of the two treatment groups. *P<0.05.
